# Prognostic value of Siglec-15 expression in patients with solid tumors: A meta-analysis

**DOI:** 10.3389/fonc.2022.1073932

**Published:** 2023-01-11

**Authors:** Kui-Ying Jiang, Li-Li Qi, Xin-Bo Liu, Yong Wang, Ling Wang

**Affiliations:** ^1^ Department of Orthopedic Oncology, The Third Hospital of Hebei Medical University, Shijiazhuang, Hebei, China; ^2^ Experimental Teaching Center, Hebei Medical University, Shijiazhuang, Hebei, China; ^3^ Department of thoracic surgery, The Fourth Hospital of Hebei Medical University, Shijiazhuang, Hebei, China; ^4^ Department of Academic Research, The Third Hospital of Hebei Medical University, Shijiazhuang, Hebei, China

**Keywords:** solid tumor, Siglec-15, prognostic biomarker, meta-analysis, immune

## Abstract

**Background:**

Siglec-15 is expressed in a variety of cancers. However, the role of Siglec-15 in the prognosis of cancer patients remains controversial. Therefore, we conducted a meta-analysis to clarify the potential prognostic value of Siglec-15 in solid tumors.

**Methods:**

The PubMed, Web of Science, Embase and CNKI databases were comprehensively searched to identify studies assessing the effect of Siglec-15 on the survival of cancer patients. Hazard ratios (HRs) with 95% confidence intervals (CIs) for overall survival (OS), progression-free survival (PFS) and disease-specific survival (DSS) from individual studies were evaluated.

**Results:**

The data from 13 observational studies consisting of 1376 patients were summarized. Elevated baseline Siglec-15 expression was significantly correlated with poor OS (pooled HR = 1.28, 95% CI: 1.05–1.56; P = 0.013). However, high Siglec-15 expression predicted a significantly better DSS (pooled HR = 0.73 (95% CI: 0.57–0.94; P = 0.015) but not PFS (pooled HR = 1.49, 95% CI: 0.46–4.87; P=0.510). In addition, high Siglec-15 expression was not associated with PD-L1 (OR=0.64, 95% CI: 0.42–0.95; P = 0.028). High Siglec-15 expression was associated with male sex (OR = 1.39, 95% CI: 1.05-1.84; P = 0.022), larger tumor size (OR = 1.896, 95% CI: 1.26-2.9; P = 0.002), and advanced tumor-node-metastasis (TNM) stage (OR = 1.84; 95% CI: 1.19-2.84; P =0.006) in solid tumors.

**Conclusions:**

This updated study suggested the expression of Siglec-15 is significantly associated with poor outcomes in human solid tumors, but further studies are needed to determine the prognostic value of Siglec-15 in solid tumors.

## Introduction

Sialic acid-binding immunoglobulin-type lectin 15 (Siglec-15) is a Siglec family member and belongs to the immunoglobulin superfamily of adhesion molecules (1). The Siglec family plays an important role in cell activation, proliferation and apoptosis and participates in the regulation of various immune responses, such as innate immunity, adaptive immunity and immune tolerance (2, 3). A type 2 constant region and an immunoglobulin variable region (IgV) can be found in the Siglec-15 extracellular structural domain (IgC2) (4). Siglec-15 is mainly expressed in human dendritic cells and macrophages, and it is highly conserved in vertebrates and functions as an immunoreceptor (5, 6). It shows mutually exclusive expression with PD-L1, suggesting that in patients who have not responded to anti-PD-1/PD-L1 therapy, therapies targeting Siglec-15 would be more beneficial. (7, 8).

Siglec-15 mRNA is abnormally overexpressed in various cancer types, such as breast cancer, cholangiocarcinoma, esophageal cancer, pancreatic adenocarcinoma, cutaneous melanoma, gastric adenocarcinoma, thyroid cancer, acute lymphoblastic leukemia, bladder cancer and endometrial cancer (9–12). Tumor cells expressing Siglec-15 may also be associated with cancer cell biological behaviors such as migration, invasion, and metastatic capacity, and ultimately affect cancer progression. (13, 14). The overexpression of Siglec-15 has been linked to a poor clinical outcome in some cancers, while it has been observed the opposite relationship in others. In osteosarcoma, endometrial clear cell carcinoma (ECCC), esophageal squamous cell carcinoma (ESCC), nasopharyngeal carcinoma (NPC) and anaplastic thyroid carcinoma (ATC), patients with high Siglec-15 expression may have a poor prognosis (13, 15–19). However, according to Chen et al., Siglec-15 positivity is associated with a better prognosis in pancreatic ductal adenocarcinoma. (PDAC) (20). There are conflicting reports on the ability of Siglec-15 to predict outcomes in various cancers.

However, no pooled analysis of Siglec-15 in predicting cancer patient prognosis has been conducted thus far. Therefore, in this article, we conducted a meta-analysis of the prognostic association of Siglec-15 overexpression with outcome in various solid tumors.

## Materials and methods

### Search strategy

**Figure 1:**
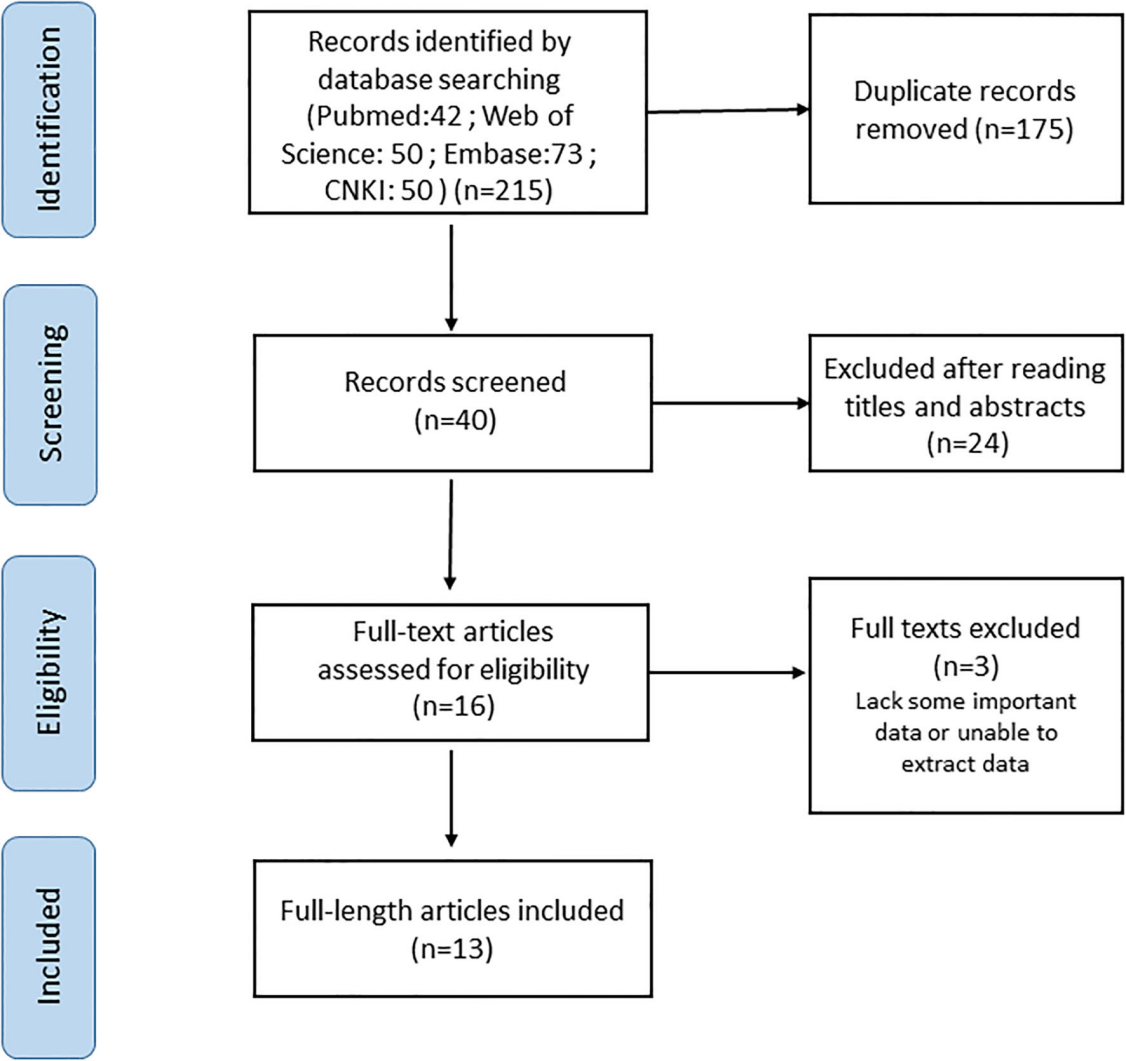
Flow chart of the selection of studies in the meta-analysis.

The PubMed, Web of Science, Embase and CNKI databases were comprehensively searched from inception until 28 November 2022. The following keywords were used: “sialic acid-binding immunoglobulin-type lectin15 OR Siglec-15 OR Siglec15” AND “cancer OR tumor OR carcinoma OR neoplasm” AND “prognosis OR survival OR outcome” (all fields). Searches were limited to human research articles published in English or Chinese. The reference lists of the retrieved studies and reviews were manually searched to identify additional potential studies. The full electronic search strategy is detailed in **Figure 1**.

### Study selection

Studies were considered eligible if they met the following initial inclusion criteria: (a) studies of humans (adults and children); (b) studies of Siglec-15 expression in tumor tissue or serum; (c) studies that linked Siglec-15 expression to survival outcomes and provided sufficient data to estimate the hazard ratio (HR) and 95% confidence interval (CI); and (d) studies of patients with solid tumors. Studies were excluded if they (a) were review articles, conference abstracts, case reports, animal studies or letters; (b) contained unpublished data; (c) focused on malignant tumors of the hematological system; or (d) lacked critical data for the pooled calculation. Two reviewers independently reviewed the full articles for eligibility.

### Data extraction

The following data were extracted by two investigators: first author’s name, publication year, country, cancer type, number of patients, sex of patients, tumor stage, detection method, cutoff value, statistical method for survival analysis, expression of Siglec-15 and PD-L1, and HRs with corresponding 95% CIs for OS, and/or progression-free survival (PFS), and/or disease-specific survival (DSS). Multivariate Cox analysis results were prioritized for inclusion if available. If HRs and CIs were not reported, they were estimated from survival curves by two investigators using the methods described by Tierney et al. (21).

### Quality assessment

The quality of each of the 13 eligible studies was evaluated independently by two investigators according to the Newcastle−Ottawa Quality Assessment Scale (NOS) (22). Scores for quality assessment ranged from 0 to 9 (9 being the best), with studies scoring 6 or higher considered high quality. Studies with a score of at least 4 were included in the subsequent pooled analysis.

### Statistical analysis

This meta-analysis was performed with Stata 15.0 (Stata Corporation, College Station, TX, USA). Pooled HR estimates with 95% CIs were used to assess the relationship between Siglec-15 expression and survival outcome. Furthermore, the Cochrane’s Q test and I^2^ statistical test were used to evaluate the heterogeneity between studies, and when heterogeneity was negligible (I^2^ < 50%), the fixed effects model (Mantel–Haenszel method) was used. Otherwise, a random effects model was used. Publication bias was assessed using Begg’s and Egger’s tests (with P < 0.05 indicating significant publication bias) (23). All P values less than 0.05 were defined as statistically significant. The HRs and 95% CIs were extracted from articles that reported only Kaplan–Meier curves using Engauge Digitizer software (version 10.8). Tierney et al. provided the method and EXCEL program for data calculation (21).

## Results

### Study characteristics

A total of 13 articles were included in our meta-analysis by using the described search strategy (**Figure 1**). A total of 1376 participants were studied to determine the relationship between Siglec-15 expression and tumor prognoses. The patients included in the study were from China, Japan and Brazil, and was diagnosed with different cancer such as gastric carcinoma, pancreatic cancer, osteosarcoma, colorectal cancer, renal cell carcinoma (RCC), ECCC, ESCC, NPC, ATC and Retroperitoneal liposarcoma (RLPS). OS was observed in eleven investigations, PFS in three, and DSS in two. Seven studies directly published HRs and 95% CIs, while the other six estimated them. The cutoff values varied among studies. The main characteristics of included studies are presented in **Table 1**.

**Table 1:** Summary of characteristics of all studies included in the meta-analysis.

Author	Year	Country	Language	Cancer	Case number	Male/Famale	Tumor stage	Follow-up (months)	No. of Siglec-15(+)	Detection method	Cut-off	Source of HR	Outcome measures
Yang et al. (24)	2021	China	English	ccRCC	150	107/43	138/12 (I+II/III+IV)	NR	73	IHC	Total staining score of Siglec-15 =positive staining rate score*staining intensity score. The positive staining rate was scored according to the percentage of positive cells: 0 (negative), 1 (1%–25%), 2 (26%–50%), and 3 (51%–100%). The staining intensity score of Siglec-15 was evaluated as 0 (negative), 1 (weak), 2 (medium), and 3 (strong), Positive: score (4-9)	SC	OS
Chen et al. (20)	2022	China	English	PDAC	263	142/121	209/54 (I+II/III+IV)	3–65	49	IHC	Positive: n ≥5% of the tumor cells expressed	Reported	PFS/DSS
Fan et al. (13)	2021	China	English	Osteosarcoma	36	19/17	16/9/11 (EnneckingI/II/III)	60	16	IHC	Total staining score of Siglec-15 =positive staining rate score*staining intensity score. The percentage of positively stained cells was scored as 1 for ≤ 33%, 2 for 33% - 66%, 3 for ≥66%, and Siglec-15 staining was scored 1 for absent/weak, 2 for moderate and 3 for strong. Positive: score (4-9)	SC	OS
Fudaba et al. (25)	2021	Japan	English	PCNSL	60	27/33	40/20 (MSKCC score2/3)	2.3-143	14	IHC	Positive: expression on≥1% of tumor cells,	Reported	OS
Chu et al. (26)	2020	China	English	peSCC	170	NR	66/85/14/9 (Tstage1/2/3/4)	200	109	IHC	0:<1 cells/high magnification field; 1: 1 to 5 cells/high magnification field; 2: 5 to 20 cells/high magnification field; 3: 20 to 50 cells/high magnification field; 4: >50 cells/high magnification field. Positive: Score > 0	Reported	DSS
Li et al. (27)	2022	China	English	PDAC	209	124/85	71/80/58(I/II/III)	NR	119	MF-IHC	NR	SC	OS/DFS
Quirino et al. (28)	2021	Brazil	English	GC	71	47/24	6/12/51/2 (Tstage1/2/3/4)	18	53	IHC	IRS = SI (staining intensity) x PP (percentage of positive cells). Staining intensity was determined as 0 is negative; 1, weak; 2, moderate; and 3, strong. The percentage of positive cells was defined as 0 is negative; 1, 10% positive cells; 2, 11-50% positive cells; 3, 51-80% positive cells; and 4, more than 80% positive cells.	SC	OS
Huang et al. (15)	2021	China	Chinese	ESCC	129	110/19	55/74 (pT 1 + 2/3+4)	60	56	IHC	NR	Reported	OS/PFS
Zheng et al. (16)	2021	China	Chinese	ECCC	27	NR	15/12(I+II/III+IV)	120	13	IHC	NR	SC	OS/PFS
Hou et al. (17)	2022	China	English	ATC	86	38/48	NR	60	44	IHC	Median was used as a cutoff point to classify these 86 THCA patients in two groups	Reported	OS
Zhao et al. (19)	2022	China	English	NPC	182	128/54	97/85(T stage1-2/3-4)	65	69	IF	NR	Reported	OS/FFS
Song et al. (18)	2022	China	English	Osteosarcoma	52	33/19	11/31/10(I/II/III)	200	16	IHC	The positive immunohistochemistry (IHC) staining percentage (0%: negative; <5%: weak positive; 5–50%: moderate positive; > 50%: intense positive) was used to judge the positivity of Siglec-15.	SC	OS
Cui et al. (29)	2022	China	English	RLPS	91	52/39	20/71(G1G2-G3)	39.7	39	IHC	The percentage of positive cells and the staining intensity determined the IRS, with scores of 0–1, 2–4, 5–8, and 9–12 evaluated as “-”, “+”, “++”, and “+++”. “-” was identified as negative, and “+”, “++”, and “+++” were identified as positive.	Reported	OS/DFS

ccRCC, clear cell renal cell carcinoma; PDAC, pancreatic ductal adenocarcinoma; OS, osteosarcoma; PCNSL, primary CNS lymphoma; peSCC, penile squamous cell carcinoma; GC, gastric cancer; ESCC, esophageal squamous cell carcinoma; ECCC, endometrial clear cell carcinoma; NPC, nasopharyngeal carcinoma; ATC, anaplastic thyroid carcinoma; RLPS, retroperitoneal liposarcoma; IHC, immunohistochemistry; IF, immunofluorescence; SC, survival curve; NR, not report; OS, overall survival; PFS, progression-free survival; DSS, disease specific survival.

### Quality assessment

The quality of each of the 13 eligible studies included in our meta-analysis was evaluated according to NOS. The quality of all included studies varied, with scores ranging from 6 to 8 (**Table 2**). As a result, all studies were included in the following analysis.

**Table 2:** Quality assessment of all studies included in the meta-analysis with Newcastle-Ottawa Scale.

Author	Year	Selection	Comparability	Outcome	Total
Yang et al. (24)	2021	☆☆	☆☆	☆☆	6
Chen et al. (20)	2022	☆☆	☆☆	☆☆☆	7
Fan et al. (13)	2021	☆☆	☆☆	☆☆☆	7
Fudaba et al. (25)	2021	☆☆☆	☆☆	☆☆☆	8
Chu et al. (26)	2020	☆☆☆	☆☆	☆☆☆	8
Li et al. (27)	2022	☆☆	☆☆	☆☆☆	7
Quirino et al. (28)	2021	☆☆	☆☆	☆☆☆	7
Huang et al. (15)	2021	☆☆	☆☆	☆☆	6
Zheng et al. (16)	2021	☆☆	☆☆	☆☆	6
Hou et al. (17)	2022	☆☆	☆☆	☆☆☆	7
Zhao et al. (19)	2022	☆☆	☆☆	☆☆☆	7
Song et al. (18)	2022	☆☆	☆☆	☆☆☆	7
Cui et al. (29)	2022	☆☆☆	☆☆	☆☆☆	8

“☆” was scored 1 point, and the finalscore was the sum of all “☆”.

### Meta-analysis results

#### Siglec-15 expression and OS

**Figure 2:**
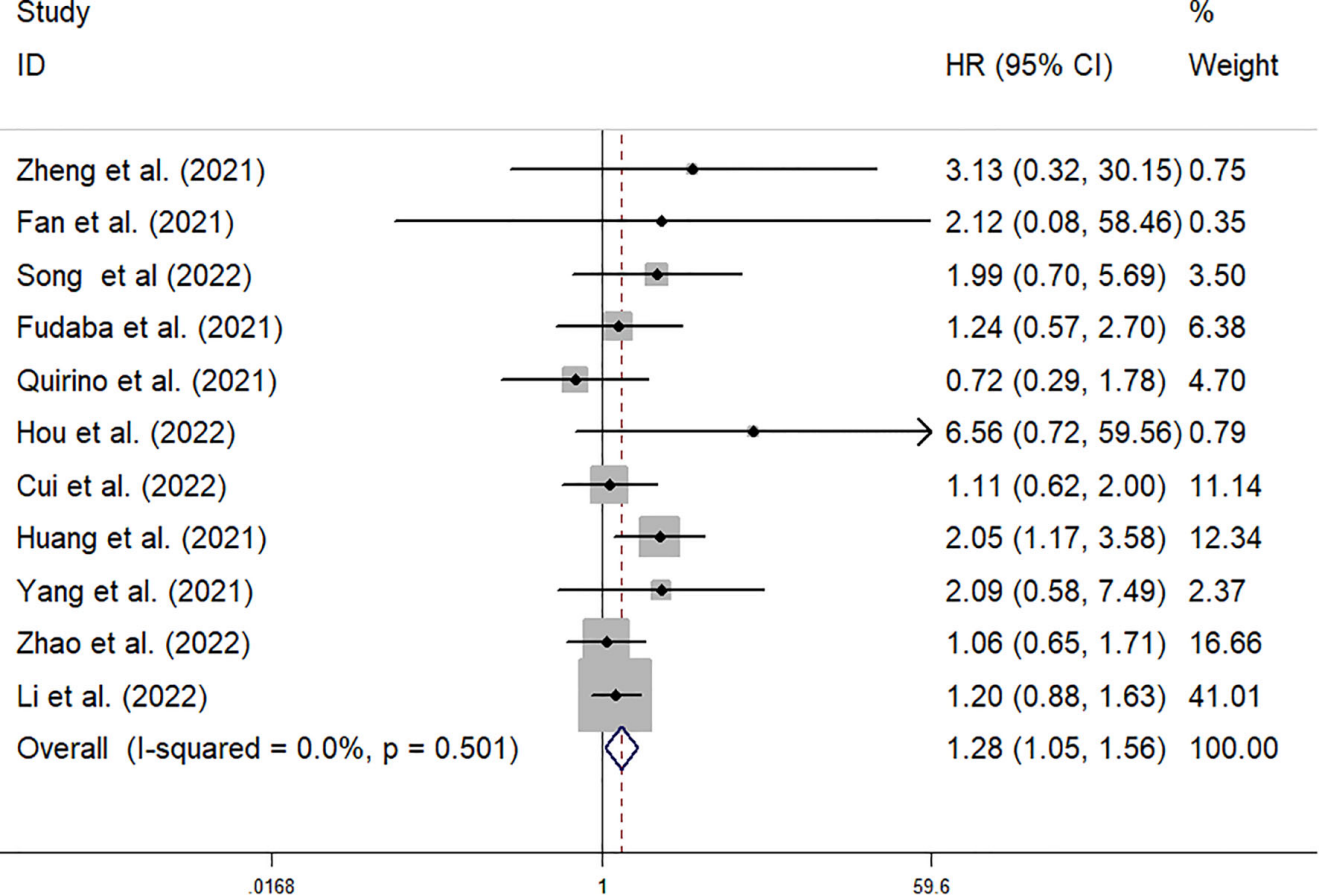
Forest plots of studies evaluating hazard ratios (HRs) of Siglec-15 for OS.

**Figure 3:**
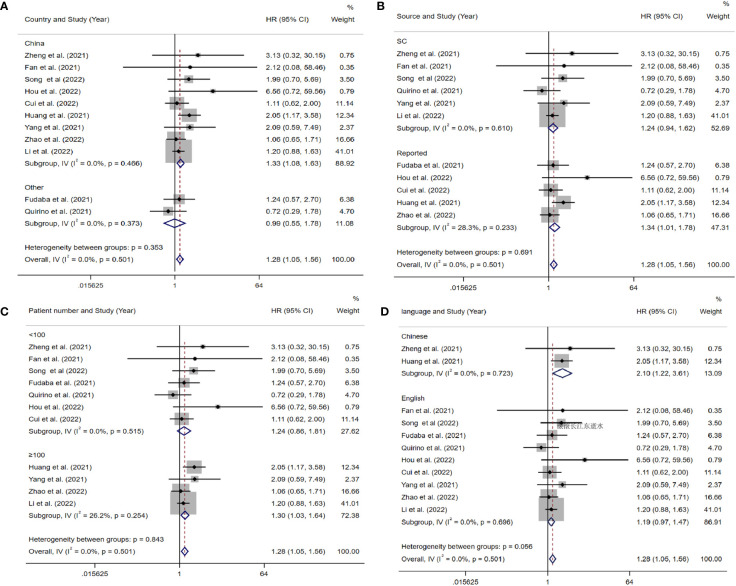
Forest plots of studies evaluating hazard ratios (HRs) of Siglec-15 for overall survival in different subgroups. **(A)** Subgroup analysis by country. **(B)** Subgroup analysis by HR source. **(C)** Subgroup analysis by patients’ number. **(D)** Subgroup analysis by language.

**Figure 4:**
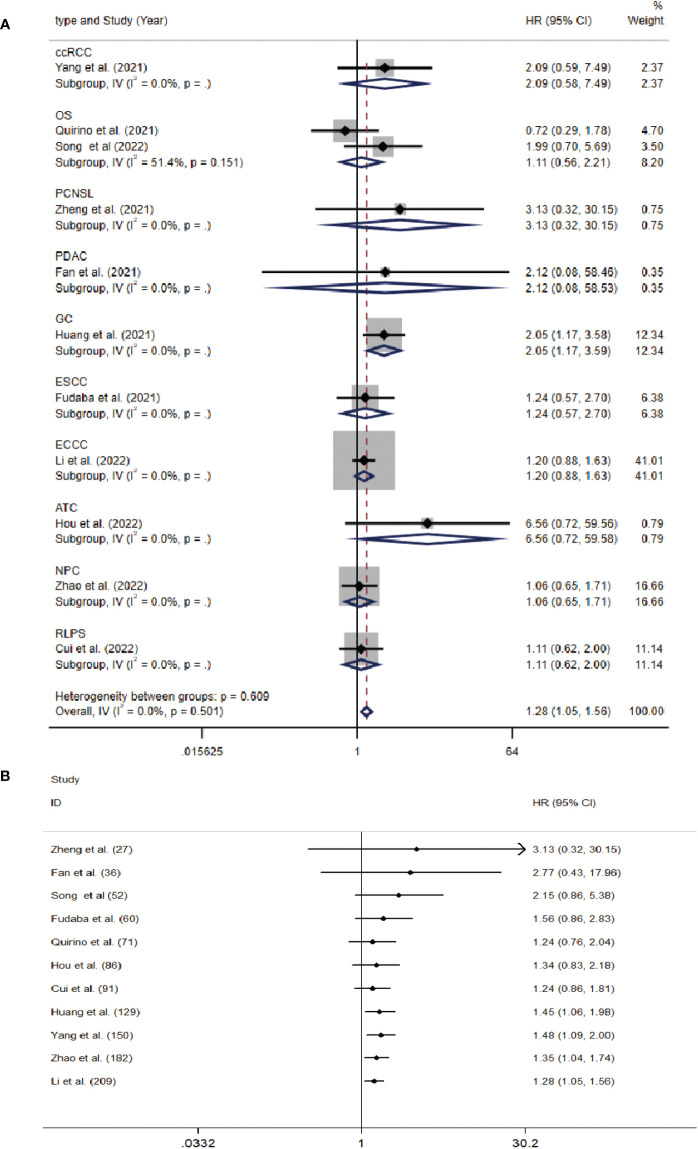
**(A)** Forest plots of studies evaluating hazard ratios (HRs) of Siglec-15 for overall survival in different subgroups of tumor type. **(B)** Cumulative meta-analysis by patients’ number.

The data from the ten included studies was suitable for OS analysis. The main results of this meta-analysis are summarized in **Table 3**. The relationship between Siglec-15 expression and OS was estimated, and the results are shown in **Figure 2**. According to the pooled analysis, Siglec-15 overexpression was significantly related with poor OS, with a combined HR of 1.28 (95% CI: 1.05–1.56; P = 0.013) (**Figure 2**). We assessed Siglec-15 expression *via* subgroup analyses based on country, source of HR, patient number (≥ 100 or not), tumor type and linguistic. High Siglec-15 expression was linked to poor OS in China (HR =1.33; 95% CI: 1.08–1.63; P = 0.008) but not in other countries (HR = 0.99; 95% CI: 0.55–1.78; P = 0.962) (**Figure 3A**). Subsequently, we found that Siglec-15 could act as a prognostic factor in groups with reported HRs (HR=1.34; 95% CI: 1.01–1.78; P = 0.045) and patient numbers ≥ 100 (HR=1.30; 95% CI: 1.03–1.64; P = 0.026) (**Figures 3B, C**). Siglec-15 also showed a predictive value in the linguistics for the Chinese groups (HR=2.10; 95% CI: 1.22-3.61; P = 0.007) and GC (HR=2.05; 95% CI: 1.17- 3.59; P = 0.012) (**Figures 3D**, **4A**). A cumulative meta-analysis showed that this evidence had been available at a sample size of 71, with additional data providing further accuracy in the point estimates but not changing the direction or magnitude of the effect (**Figure 4B**).

**Table 3:** Pooled HRs for overall survival and subgroup analysis of Siglec-15 expression.

Outcome Subgroup	No. of study	No. of patients	HR (95%CI)	p value (I^2^)
OS	11	1093	1.28 (1.05, 1.56)	0.013 (0%)
Country				
China	9	1006	1.33 (1.08, 1.63)	0.008(0%)
Other	2	87	0.99 (0.55, 1.78)	0.962(0%)
Source of HR				
Reported	7	548	1.34 (1.01, 1.78)	0.045 (28.3%)
SC	6	545	1.24 (0.94, 1.62)	0.125 (0%)
Sample size				
≥100	6	670	1.30 (1.03, 1.64)	0.026(26.2%)
<100	7	423	1.24 (0.86, 1.81)	0.254(0%)
Tumor type				
ccRCC	1	150	2.09 (0.58, 7.49)	0.256 (0%)
OS	2	88	1.11 (0.56, 2.21)	0.761 (51.4%)
PCNSL	1	60	3.13 (0.32, 30.15)	0.324 (0%)
PDAC	1	209	2.12 (0.08, 58.53)	0.656 (0%)
GC	1	71	2.05 (1.17, 3.59)	0.012 (0%)
ESCC	1	129	1.24 (0.57, 2.70)	0.586 (0%)
ECCC	1	27	1.20 (0.88, 1.63)	0.248 (0%)
ATC	1	86	6.56 (0.72, 59.58)	0.095 (0%)
NPC	1	182	1.06 (0.65, 1.71)	0.821 (0%)
RLPS	1	91	1.11 (0.62, 2.00)	0.733 (0%)
Language				
English	9	937	1.19 (0.97, 1.47)	0.103 (0%)
Chinses	2	256	2.10 (1.22, 3.61)	0.007 (0%)

#### Siglec-15 expression and PFS

**Figure 5:**
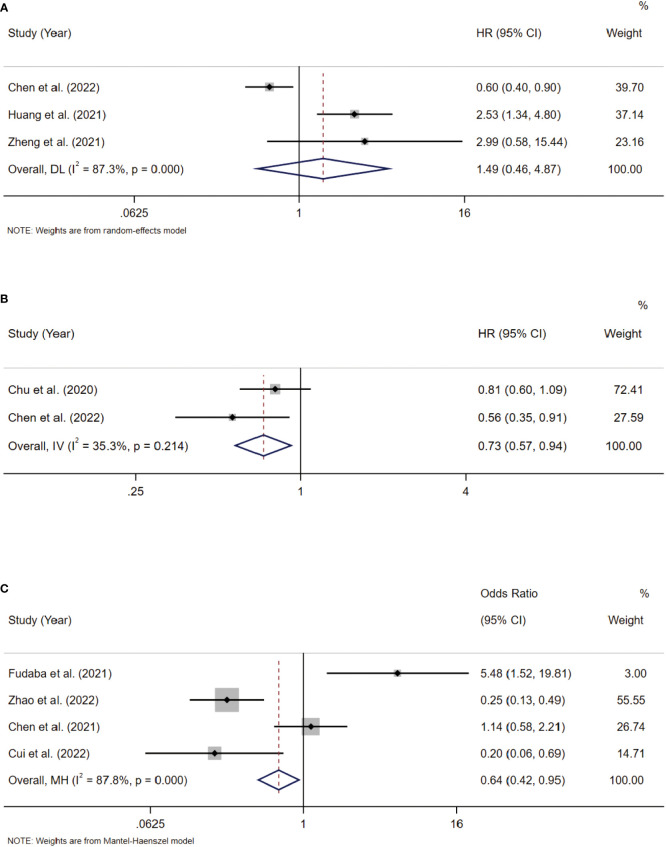
**(A)** Forest plots of studies evaluating hazard ratios (HRs) of Siglec-15 for PFS. **(B)** Forest plots of studies evaluating hazard ratios (HRs) of Siglec-15 for DSS. **(C)** Correlation between Siglec-15 and PD-L1 expression in solid tumors.

Three included studies provided suitable data for PFS analysis. According to the combined study, Siglec-15 overexpression was not significantly linked with poor PFS, with a combined HR of 1.49 (95% CI: 0.46–4.87; P = 0.510) (**Figure 5A**).

#### Siglec-15 expression and DSS

Two included studies provided suitable data for DSS analysis. According to the combined study, Siglec-15 overexpression was significantly linked with better DSS, with a combined HR of 0.73 (95% CI: 0.57–0.94; P = 0.015) (**Figure 5B**).

#### Coloration between Siglec-15 and PD-L1 expression

Blocking PD-L1 is an important strategy for the response to checkpoint blockade therapy, but its effectiveness is limited (30). The expression of Siglec‐15 and PD‐L1 are mutually exclusive in lung adenocarcinoma (31), showing that Siglec-15 has a close interaction with PD-L1. Thus, we analyzed the expression of Siglec-15 and PD-L1 in solid tumors. Three included studies provided suitable data for PD-L1 expression. According to pooled analysis, Siglec-15 overexpression was negatively correlated with PD-L1 expression, with a combined odds ratio (OR) of 0.64 (95% CI: 0.42–0.95; P = 0.028) (**Figure 5C**).

#### Association between Siglec-15 expression and clinicopathologic parameters

**Figure 6:**
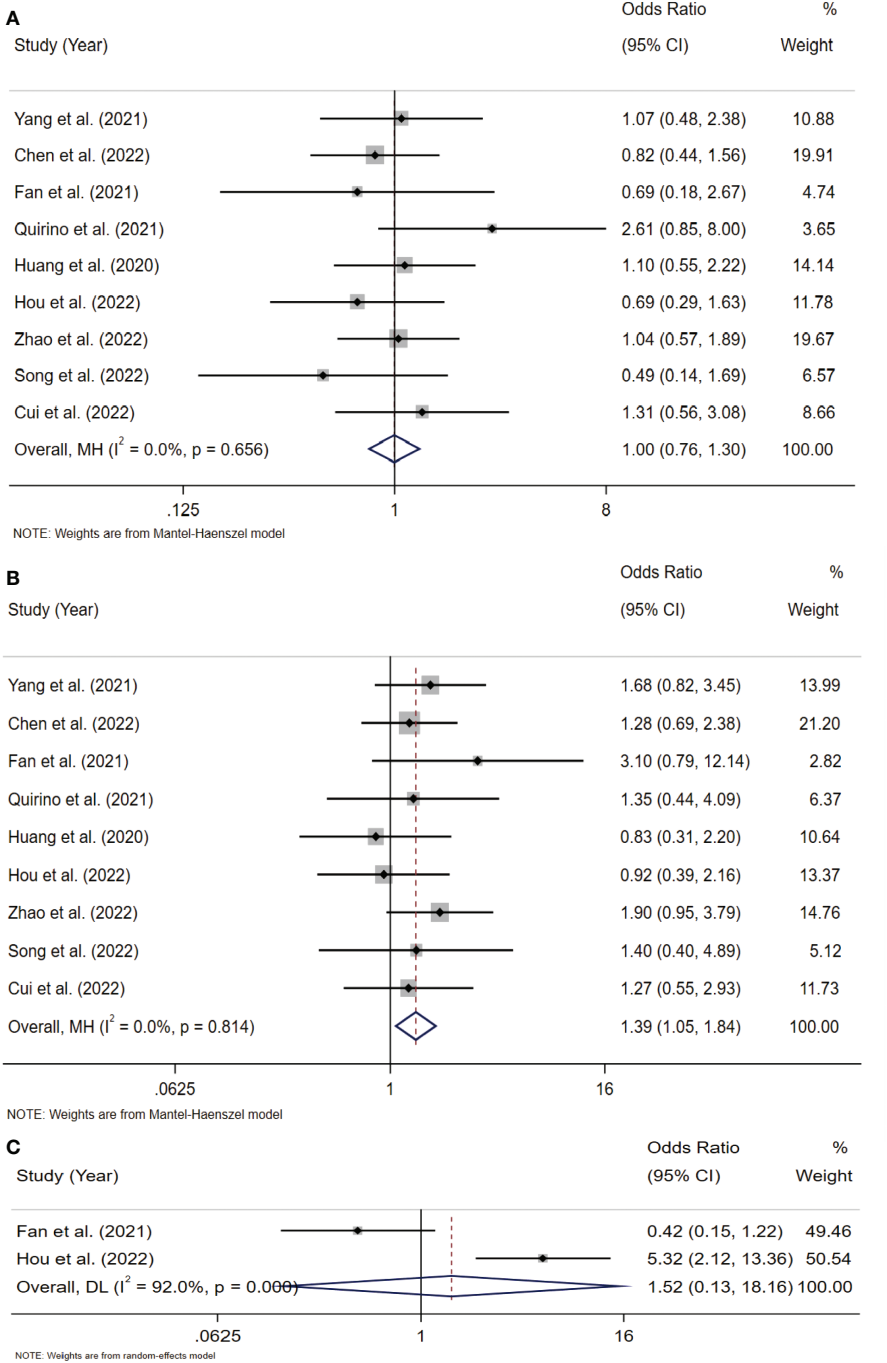
Forest plots of Siglec-15 expression and clinicopathological features in solid tumor. **(A)** Age (> 50 years old *vs*. < 50 years old); **(B)** Gender (male *vs*. female); **(C)** distant metastasis.

**Figure 7:**
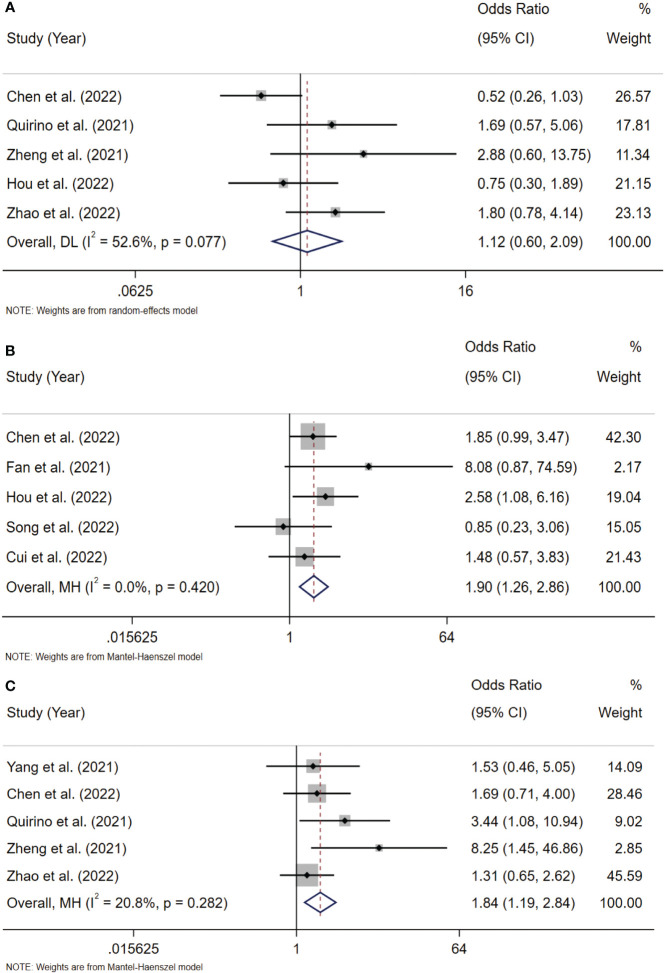
Forest plots of Siglec-15 expression and clinicopathological features in solid tumor. **(A)** LNM (Yes *vs*. No); **(B)** tumor size (>5cm *vs*.<5 cm); **(C)** TNM stage (III–IV *vs*. I–II). LNM, lymph node metastasis; TNM, tumor-node-metastasis.

To analyze the association between Siglec-15 and the clinicopathological characteristics of solid tumor patients, we further analyzed the results of studies stratified by age, sex, tumor size, lymph node metastasis (LNM), tumor-node-metastasis (TNM) stage, and distant metastasis. Notably, the obtained results indicated that high Siglec-15 expression was significantly correlated with male sex (OR= 1.39; 95% CI: 1.05-1.84; P = 0.022) (**Figure 6B**), larger tumor size (OR = 1.90; 95% CI: 1.26-2.86; P = 0.002) (**Figure 7B**), and advanced TNM stage (OR = 1.837; 95% CI: 1.187-2.843; P =0.006) (**Figure 7C**). However, there was no statistically significant relationship between Siglec-15 expression and age (OR = 0.996; 95% CI: 0.761-1.303; P = 0.977), distant metastasis (OR = 1.52; 95% CI: 0.13-18.16; P = 0.74) or LNM (OR = 1.12; 95% CI: 0.60-2.09; P = 0.72) (**Figures 6A, C**, **7A**). The detailed data are listed in **Table 4**.

**Table 4:** Association between Siglec-15 and other clinicopathologic parameters.

Outcome or subgroup	Studies	Participants	Odds ratio (95% CI)	P value	Model	Heterogeneity	
						*P* value	I^2^
Age	9	1059	0.996 (0.761, 1.303)	0.977	Fixed	0.656	0.0%
Gender	9	1059	1.391 (1.050, 1.843)	0.022	Fixed	0.814	0.0%
Tumor Size	5	528	1.896 (1.257, 2.859)	0.002	Fixed	0.42	0.0%
LNM	5	629	1.120 (0.600, 2.090)	0.722	Random	0.077	52.6%
TNM	5	693	1.837 (1.187, 2.843)	0.006	Fixed	0.282	20.8%
Distant Metastasis	3	299	1.522 (0.127, 18.164)	0.74	Random	<0.001	92.0%

### Sensitivity and publication bias analyses

**Figure 8:**
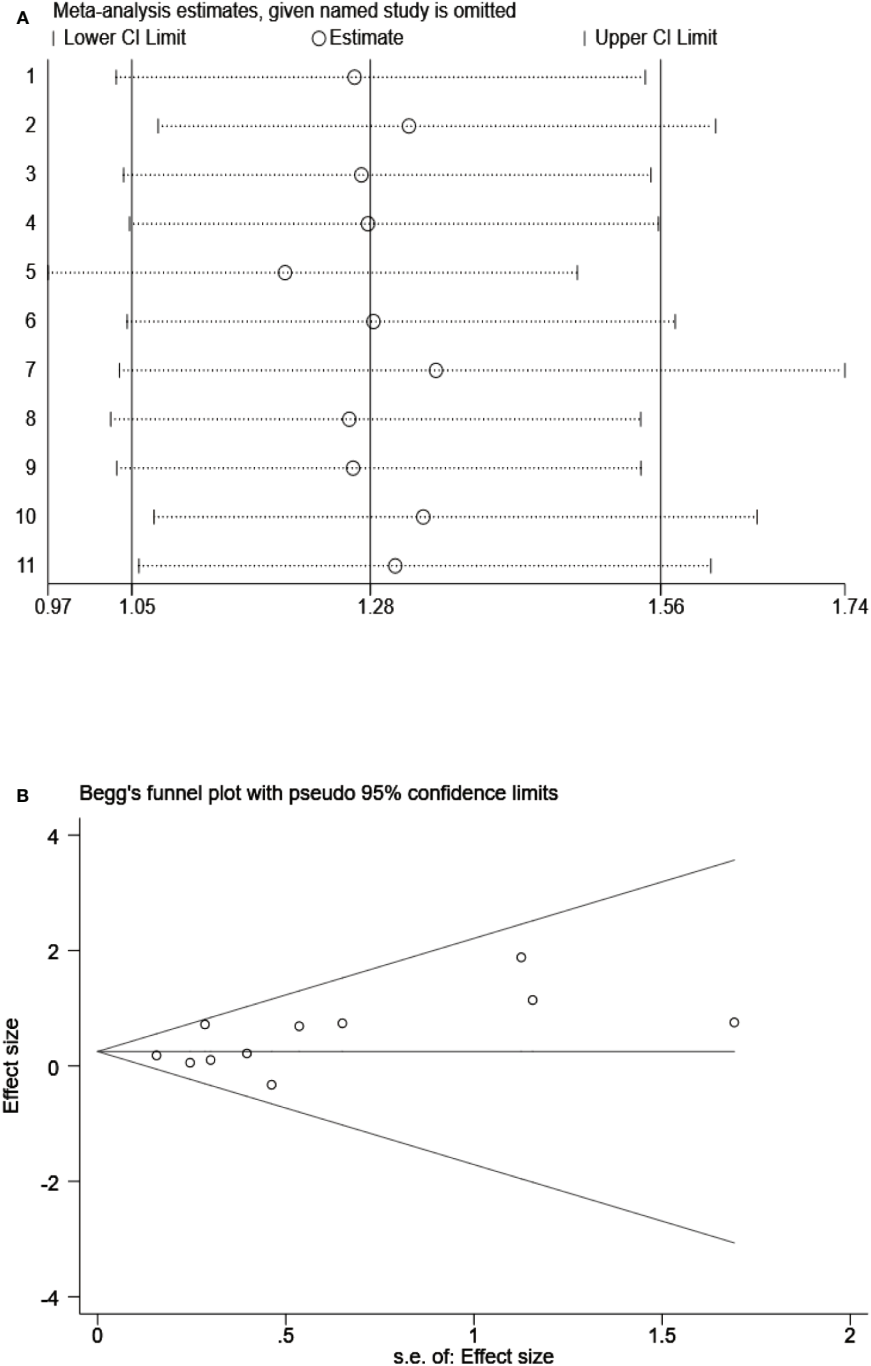
**(A)** Sensitivity analysis on the all included studies between Siglec-15 expression and OS. **(B)** Begg’s funnel plots of publication biases on the all included studies between Siglec-15 expression and OS.

To examine the stability of our results, we performed sensitivity analysis by using a random effects model and found that the results were not significantly influenced by any single study (**Figure 8A**). We also assessed the publication bias of all included studies using funnel plots and Egger’s and Begg’s tests (**Figure 8B**). The shape of the funnel plots for OS was nearly symmetrical, indicating that there was no statistically significant difference, as validated by Begg’s test (P = 0.276) and Egger’s test (P = 0.131). Thus, in these included studies, there was no evidence of significant publication bias.

## Discussion

Siglecs are cell-surface immunoglobulin-like lectins that bind sialylated glycans and are involved in a variety of physiological processes, such as the regulation of immune cell activation, proliferation and apoptosis (2, 32). Siglec-15 is a member of the Siglec family and is present in both neoplastic cells and tumor-associated stromal cells (31). Recently, Siglec-15 has been identified as a novel immune suppressor and has a role as an immune checkpoint that is not dependent on the well-known PD-L1/PD-1 pathway (33, 34). A phase I/II clinical trial of a Siglec‐15 inhibitor (NC318) in solid tumors is ongoing (NCT03665285).

In this study, we conducted a pooled analysis to explore the prognostic role of Siglec-15 in 1376 patients with various cancers from 13 studies. As far as we know, this is the first meta-analysis to focus on the correlation between Siglec-15 expression and the clinical outcomes of cancer patients. Notably, this is also the first time that the correlation between the expression of Siglec-15 and PD-L1 has been evaluated.

Siglec-15 is highly expressed in the majority of cancer types. Our results indicated that high expression of Siglec-15 is associated with poorer OS and might act as an independent risk factor. However, the expression of Siglec-15 was significantly associated with better DSS and not significantly associated with PFS. This may be influenced by the different types of cancer. In the majority of cancer types, high expression of Siglec-15 was associated with poor prognosis, but there was an opposite conclusion in the study of Chen et al, which revealed that Siglec-15 positivity was associated with better PFS and DSS in PDAC (20). In the subgroup analysis, Siglec-15 showed the inconsistent prognostic effects in different country. The expression of Siglec-15 was correlated with poor OS in China but was not in other country. The similar phenomenon was observed in subgroup analysis based on patient number as Siglec-15 in studies with ≥100 patients linked with poor OS, while it was not statistically significant in studies with <100 patients. Siglec-15 also showed a predictive value in GC and the linguistics for the Chinese groups. This difference may be due to different geographical and demographical features of various regions. Finally, we are confident that the results are reliable since most studies showed a low risk for bias.

Overexpression of Siglec-15 has been associated with a variety of cancers, including bladder cancer, breast cancer, cholangiocarcinoma, thyroid cancer, esophageal carcinoma, osteosarcoma, head and neck squamous cell carcinoma, kidney cancer, hepatocellular carcinoma, gastric cancer, and endometrial carcinoma (9, 13, 35). Yang et al. found that Siglec-15 was positively associated with poorer prognosis in ccRCC (24). In osteosarcoma, high expression of Siglec-15 was related with pulmonary metastasis and predicted poor prognosis (13). Quirino et al. found that Siglec-15 expression was not related to OS or recurrence-free survival (RFS) in gastric cancer (28), and a similar phenomenon was observed in early non-small cell lung cancer (NSCLC) in which Siglec-15 was not associated with OS (36). In contrast, high Siglec-15 expression was linked to better prognosis in PDAC and penile squamous cell carcinoma (peSCC) (20, 26). This can be as a result of the different features of various cancers as well as the differences in sample sizes or measurements. Siglec-15 was a strong negative predictor of OS in cancer patients according to our pooled results, suggesting that Siglec-15 is a potent prognostic predictor for survival outcomes in patients.

In addition to tumor cells, Siglec-15 can also be expressed in tumor-associated macrophages (TAMs) (37). Li et al. revealed that PDAC patients with Siglec-15^+^TAMs had poorer OS and disease-free survival (DFS), while neither OS nor DFS was distinguishable between patients in the tumor-Siglec-15 low group and tumor-Siglec-15 high group (27). Similarly, in primary central nervous system lymphoma (PNCSL), the median OS was significantly longer in patients with Siglec-15-positive peritumoral macrophages than in patients with Siglec-15-negative peritumoral macrophages. However, both the positive and negative expression of Siglec-15 on tumor cells and intratumoral macrophages had no influence on OS (25). In our study, we only analyzed the data of Siglec-15 expressed in tumors and revealed that Siglec-15 expression in tumors was not related to improved PFS. Thus, this may explain the negative results of PFS.

Siglec-15 is highly homologous to the B7 gene family, of which PD-L1 is a member (31). Siglec-15 might regulate immune escape from tumors, along with PD-L1. For patients who have not responded to anti-PD-1 medication, targeting Siglec-15 may be a potential therapeutic alternative. In our study, Siglec-15 overexpression was negative linked with PD-L1 (OR = 0.64, 95% CI: 0.23–4.94, P = 0.936), suggesting that it may be a potential immunotherapy target and may expand the therapeutic benefit groups of patients with solid tumors. Future research is required to validate the prognostic significance of Siglec-15 and PD-L1 and to investigate their action mechanisms in tumor. Then, we analyzed the relationship between Siglec-15 and some clinical features, including age, sex, tumor size, LNM, TNM stage and distant metastasis. Higher Siglec-15 expression was associated with male sex and predicted larger tumor sizes and advanced clinical stages. There was no statistically significant relationship between Siglec-15 expression and age, LNM, or distant metastasis. The above results show that Siglec-15 may affect the prognosis of patients with solid tumors, suggesting a poor prognosis. Further well-designed, large cohort studies are needed to corroborate this trend.

Our analysis was subject to several important limitations. First, the limitations of the study design included those inherent in any meta-analysis, such as heterogeneity among studies caused by differences in study populations, lack of a unified cutoff value for Siglec-15 expression (with various cutoff values among the eligible studies) and lack of a multivariate control. Second, publication bias was undetected but cannot be excluded because the analysis was subject to publication bias and relied on summary data. Finally, several HRs were not reported in some studies and had to be calculated using data extracted from survival curves, resulting in slight statistical errors. These limitations may affect the efficiency of Siglec-15 as a prognostic biomarker. Although the clinical significance of Siglec-15 in different cancers has been demonstrated, the different effects of Siglec-15 on different tumors and its different receptors remain to be determined. More studies investigating Siglec-15 expression and its relationship with prognostic outcomes are needed.

## Conclusions

Our findings showed that Siglec-15 expression is related with worse OS in human solid tumors, suggesting that Siglec-15 expression could be a valuable indicator for the diagnosis and prognosis of solid tumors. Given the limits of our investigation, this result should be approached with caution. Targeting Siglec-15 may provide a potential treatment option for patients with solid tumors. However, further large-scale and comprehensive research is required to determine the significance of Siglec-15 in cancer prognosis and targeted therapy.

## Data availability statement

The original contributions presented in the study are included in the article/supplementary material. Further inquiries can be directed to the corresponding author.

## Author contributions

K-YJ: Formal analysis, Software, Data curation, Validation, Writing- Original draft preparation. L-LQ: Data curation, Writing - original draft. XB-L: Writing - original draft. YW: Writing - original draft. LW: Writing - review and editing, Funding acquisition. All authors contributed to the article and approved the submitted version.
